# A novel method for objectively classifying sequential emotion within dreams: a proof-of-concept pilot study

**DOI:** 10.3389/fpsyg.2024.1393913

**Published:** 2024-09-18

**Authors:** Mariza van Wyk, Mark Solms, Gosia Lipinska

**Affiliations:** UCT Sleep Sciences, Department of Psychology, University of Cape Town, Cape Town, South Africa

**Keywords:** emotion regulation, emotions in dreams sentiment analysis, dream emotional intensity, objective dream ratings, classifying dream emotions, trapezoid rule, area under the curve

## Abstract

Traditionally, emotions in dreams have been assessed using subjective ratings by human raters (e.g., external raters or dreamers themselves). These methods have extensive support and utility in dream science, yet they have certain innate limitations due to the subjective nature of the rating methodologies. Attempting to circumvent several of these limitations, we aimed to develop a novel method for objectively classifying and quantifying sequential (word-for-word) emotion within a dream report. We investigated whether sentiment analysis, a branch of natural language processing, could be used to generate continuous positive and negative valence ratings across a dream. In this pilot, proof-of-concept study, we used 14 dream reports collected upon awakening following overnight polysomnography. We also collected pre- and post-sleep affective data and personality metrics. Our objectives included demonstrating that (1) valence ratings derived from sentiment analysis (Valence Aware Dictionary for sEntiment Reasoning [VADER]) could be used to visualize (plot) positive and negative emotion fluctuations within a dream, (2) how the visual properties of emotion fluctuations within a dream (peaks and troughs, area under the curve) can be used to generate novel “emotion indicators” as proxies for emotion regulation throughout a dream, and (3) these emotion indicators correlate with sleep, affective, and personality variables known to be associated with dreaming and emotion regulation. We describe 6 novel, objective dream emotion indicators: Total number of Peaks, total number of Troughs, Positive, Negative, and Overall Emotion Intensity (composites from an “area under the curve” method using the trapezoid rule applied to the peaks and troughs), and the Emotion Gradient (a polynomial trendline fitted to the emotion fluctuations in the dream chart). The latter signifies the overall direction of sequential emotion changes within a dream. Results also showed that ⅚ emotion indicators correlated significantly with at least one existing sleep, affective, or personality variable known to be associated with dreaming and emotion regulation. We propose that the novel emotion indicators potentially serve as proxies for emotion regulation processes unfolding within a dream. These preliminary findings provide a methodological foundation for future studies to test and refine the method in larger and more diverse samples.

## 1 Introduction

Traditionally, content and emotion in dreams have been assessed through three main methods with varying degrees of subjectivity associated with each method: (1) self-ratings by the dreamer themselves, (2) ratings by a blind external judge, (3) external ratings based on a classification system developed by Hall and van de Castle (1966; Röver and Schredl, 2017; Zadra and Domhoff, 2011). These methods have widespread support in dream science, yet it has obvious limitations associated with the subjective nature of these ratings (Röver and Schredl, 2017; Schredl and Doll, 1998; Schredl, 2010a; Sikka et al., 2014). Some of the limitations include significant disparities across these methods with regard to the presence and/or intensity of emotions in dreams, over- and/or under-estimation of positive and negative emotions, incongruencies in ratings of explicitly mentioned emotions versus implicit/experienced emotions (the latter being more likely to be identified by self-raters), as well as discrepancies in the emotion/content classification systems/scales used.

Although there are many advantages to obtaining subjective data, objective approaches provide advantages of their own. Sentiment analysis, a semantic analytic technique and a branch of natural language processing (NLP), is a powerful tool used to analyze the emotional properties of texts. It works by classifying and quantifying positive, negative, and neutral emotions detected in texts. Depending on the method used, sentiment analysis assigns a categorical or continuous valence rating to the overall text, sentences in the text, or individual words in the text. Encouragingly, sentiment analysis has recently been successfully employed to analyze emotion in dream reports (Yu, 2022). More specifically, one of the methods used in the study by Yu (2022) is called the Valence Aware Dictionary for sEntiment Reasoning (VADER)—an automated software program that analyses textual data based on an established lexicon, coupled with annotated lexical features. As an objective approach to rating emotions in dreams, the VADER program was described by the authors as a reliable and effective sentiment analysis tool. It is proposed to complement existing methods in the following ways: (1) detecting subtle, indirect affective feelings by considering the tone of every word used, (2) circumventing inter-rater discrepancies due to its automated nature, and (3) generating continuous (as opposed to categorical) classifications of emotion/valence. This yields a metric that captures the emotional intensity of dream experiences in an objective manner. Therefore, the three main properties of the VADER program enable one to objectively track sequential (word-for-word) emotion intensity within a dream by generating continuous positive and negative valence ratings. This method circumvents several of the limitations/biases inherent to strictly subjective approaches to rating emotions in dreams. Additionally, this method also overcomes some of the limitations present in other forms of semantic analysis, such as the dictionary-based approach, which determines the word frequency count for specific categories, such as positive or negative words. This approach is limited in that it does not take context into account (for example, what was said before or after a particular counted word) and does not measure emotion continuously, as it progresses through the dream (Elce et al., 2021).

### 1.1 Rationale and aims

Based on current and ongoing limitations evident in dream rating, we had the following main aim: to develop an objective method to classify and quantify emotion in a sequential (word-for-word) manner within individual dream reports. With this exploratory approach we aimed to provide preliminary evidence for the proposition that sequential emotion fluctuations could serve as objective indicators of emotional processes unfolding within a dream. We set out to achieve this by using sentiment analysis to generate sequential emotional valence ratings throughout a dream. We then plotted and visualized these valence ratings and applied specific statistical and mathematical techniques to the peak and trough characteristics of the chart to create a range of “emotion indicators.” To validate whether these indicators are effective in quantifying dream emotion, we correlated them with external variables that are known to be related to dreaming and/or emotion regulation. These external variables fell into a number of categories that included polysomnography-derived parameters (e.g., number of awakenings and sleep stage distribution, classified as a “sleep” variable), along with pre- and post-sleep affective data (which we classified as “state” variables), and, finally, personality characteristics (which we classified as “trait” variables). We aimed to link, in a preliminary fashion, these external variables with the dream emotion indicators. In summary, we had the following objectives:

1. We aimed to show that sentiment analysis could be used to classify, quantify, and visualize positive and negative emotion fluctuations within a dream;
2. The visual properties of emotion fluctuations within a dream (peaks and troughs) can be used to generate novel “emotion indicators”; and:
3. The various emotion indicators are correlated with sleep, state, and trait variables.

Here, we present our new method, and accompanying preliminary findings, as a proof-of-concept (POC) study—a first step in developing a novel method for objectively classifying sequential emotion within a dream. We propose that the emotion indicators could serve as potential proxies for emotion regulatory processes during dreaming. With these preliminary results we aim to provide a methodological foundation for future studies to test and refine the method employed in the current study in larger and more diverse samples.

## 2 Methods

### 2.1 Participants

This pilot study contained dream reports from 14 healthy university students. The sample used in this study is a subset of a sample recruited for a larger study which investigated sleep architecture, memory, and affect in relation to dream recall frequency (DRF). Screening for the larger study occurred in two phases: the first phase consisted of an online survey, and the second phase consisted of a face-to-face clinical interview. During the online screening phase, participants completed questions related to demographics, medical and psychiatric history, sleep quality, unusual sleep experiences (e.g., sleep paralysis), medication use, and DRF.

Participants were excluded from participation if they met any of the following criteria: (a) were below the age of 20 or over the age of 40, (b) had any medical or neurological condition that could affect the outcomes of the study, (c) had a current and/or past history of any sleep disorder, (d) had a current and/or past history of any psychiatric disorder, (e) used sleeping pills, sedative medication, psychoactive medication or any other medication that might affect sleep or dreaming, (f) had a past and/or current history of alcohol or substance abuse or dependence, (g) were currently pregnant, or (h) had below-average cognitive ability. Literature shows that these factors have an independent relationship with sleep and/or dreaming and can affect study outcomes (Lee, 1998; Blackman, 2000; Irwin et al., 2000; Nielsen and Stenstrom, 2005; Schredl, 2010b; Pagel, 2010; Schredl et al., 2013; Skancke et al., 2014).

All eligible participants underwent overnight polysomnography (PSG) on two non-consecutive nights. The qualifying/inclusion criterion for the current study was participants reporting a dream upon awakening following the second (testing) night at the sleep laboratory. This was to ensure that we would be able to correlate dream emotion indicators with the external, validated (1) sleep (PSG-derived) variables, (2) affective (state), and (3) personality (trait) variables.

With regard to the demographic characteristics of the final sample for the current study, 57% of participants identified as women and 43% of the sample identified as men. The average age was 21.93 with a range of 20–27.

### 2.2 Study procedure

All study procedures complied with the Declaration of Helsinki. Ethical clearance from the relevant ethical review boards of both the Department of Psychology and the Faculty of Humanities was obtained prior to recruitment commencing. All participants included in this study completed an in-person informed consent form.

#### 2.2.1 Online screening

The (a) *Michigan Alcoholism Screening Test* (MAST) (Selzer, 1971) was used to exclude participants with alcohol dependence (MAST >4), (b) the *Pittsburgh Sleep Quality Index* (PSQI) (Buysse et al., 1989) was used to exclude participants with poor sleep quality (PSQI >5), and (c) the *Beck Depression Inventory*, 2nd Edition (BDI-II) (Beck et al., 1996) was used to exclude participants presenting with depressive symptoms (BDI-II > 13).

#### 2.2.2 Face-to-face screening

The *Mini International Neuropsychiatric Interview* (Version 5.0.0; MINI) (Sheehan et al., 1998) was used to exclude participants with any major psychiatric disorders contained in the Diagnostic and Statistical Manual of Mental Disorders (DSM-V) (American Psychiatric Association, 2013). These disorders include depression, bipolar disorder, posttraumatic stress disorder, alcohol abuse/dependence, substance abuse/dependence, and obsessive-compulsive disorder. This measure was also used to cross-validate results obtained via the MAST and BDI-II. More specifically, participants who did not meet the criteria for alcohol abuse/dependence and/or depression on the online screening measures but did meet the criteria for these conditions on the MINI were excluded from participation.

### 2.3 External experimental measures

#### 2.3.1 Most recent dream form

Participants were asked to complete a Most Recent Dream Form (Domhoff and Schneider, 1998) upon awakening (conclusion of the sleep study). This form asks participants to write down the last dream they can remember (irrespective of when it occurred) in as much detail as possible. This form was used as a criterion for inclusion in the current study—only participants who recalled a dream from the night before upon awakening in the sleep lab, were included in the final sample.

#### 2.3.2 Polysomnography

Two 16-channel Nihon Kohden Neurofax EEG900 electroencephalographs that were adapted for research recorded objective measures of sleep. The PSG included electroencephalography (EEG) which measured brain activity, electrooculography (EOG) which monitored eye movements, chin electromyography (EMG) which monitored muscle tone, and electrocardiography (ECG) which measured heart rate.

A bipolar montage was used with the following bipolar derivations: F3-C3, C3-P3, P3-O1, and F4-C4, C4-P4, P4-O2. This was combined with a referential montage utilizing F3-A2, C3-A2, O1-A2, and F4-A1, C4-A1, O2-A1 derivations. A combination approach was chosen in order to ensure the integrity of the records. Standard filters for sleep recordings were used for the EEG and EOG (0.5–35 Hz), EMG (10–70 Hz), and ECG (1–70 Hz) as recommended by AASM guidelines (American Academy of Sleep Medicine, 2015).

The following external experimental variables were derived from PSG: (1) the proportion of stage 1 non-REM sleep (N1%), (2) the proportion of stage 2 non-REM sleep (N2%), (3) the proportion of stage 3 non-rapid eye movement sleep (N3%), (4) the proportion of REM sleep (REM%), total number of awakenings across the night, the number of awakenings from N1, N2, N3, and REM sleep, as well as the proportion of time spent awake after sleep onset (WASO%).

### 2.4 Affective/state measures

The measure we used to assess parameters related to positive and negative affect is the *Positive and Negative Affect Schedule—Expanded Version* (PANAS-X) (Watson and Clark, 1994). This questionnaire consists of 60 items measuring 11 specific affects: Fear, Sadness, Guilt, Hostility, Shyness, Fatigue, Surprise, Joviality, Self-Assurance, Attentiveness, and Serenity in the broad context of positive and negative affect. Participants completed the questionnaire in its entirety on the evening before going to sleep on the second (testing night) at the sleep laboratory, as well as the following morning.

We used the “general positive affect” and “general negative affect” composites derived from the evening and morning PANAS to generate the following affective (state) variables to include in our study: (1) Night-time Positive Affect, (2) Night-time Negative Affect, (3) Morning Positive Affect, (4) Morning Negative Affect, (5) overnight percentage change in positive affect, and (6) overnight percentage change in negative affect. A higher score for variable 5 indicates a larger *positive* increase in overnight positive affect (more positive affect the following morning), while a higher score for variable 6 indicates a larger increase in overnight *negative* affect (more negative affect the following morning).

### 2.5 Personality/trait measures

The first personality/trait measure, the *Ten-Item Personality Inventory* (TIPI) (Gosling et al., 2003), is a short measure of personality based on the “Big Five” personality dimensions. These dimensions include “extroversion,” “agreeableness,” “openness to experience,” “conscientiousness,” and “neuroticism” (its converse being “emotional stability”). This short measure was chosen in order to limit the testing burden on participants in the larger study.

The second personality/trait measure we used was the *Boundary Questionnaire—Shortform* (BQ-Sh) (Rawlings, 2001). This measure is based on the original Boundary Questionnaire developed by Hartmann (1989). By means of factor analysis, the BQ-Sh is a short version (46 items) empirically derived from the full (145 items) version. The items in the questionnaire relate to how “permeable” a person’s boundary is to external influences. In other words, people with thin boundaries often demonstrate a fluid sense of self, they tend to be sensitive, vulnerable, and sometimes fail to distinguish between fantasy and reality. Conversely, individuals with “thick” boundaries have a distinct sense of self separate from others, can clearly distinguish between fantasy and reality, and are often guarded, and meticulously careful in their actions (Hartmann, 1989; Rawlings, 2001). A higher score on the Boundary Questionnaire is indicative of “thinner” boundaries, i.e., having a more permeable sense of self in relation to the external world.

### 2.6 Emotion indicators

We developed several novel emotion indicators using the following procedure: generating word-for-word emotional valence ratings throughout the dream via sentiment analysis, (2) applying a “sliding window method” to these valence ratings to control for potential confounds, (3) visualizing the word-for-word (sequential) valence ratings for each dream by plotting the ratings, (4) applying statistical and mathematical techniques to the peak (representing positive emotion) and trough (representing negative emotion) characteristics of the chart to generate the different emotion indicators, (5) validating the emotion indicator variables by correlating them with existing measures/variables known to be associated with dreaming and emotion regulation. Below we describe each step in more detail.

### 2.7 Sentiment analysis

We employed sentiment analysis, a branch of natural language processing (NLP), that classifies the components of a text as negative, neutral, or positive based on an established lexicon. More specifically, we tested our method by using the VADER package in R statistical software (version 2023.09.0 + 463). The VADER package was recently applied to dream emotion analysis, with some promising results (Yu, 2022). Regarding the analytic process, the software assigns emotion (valence) ratings to each word in the dream report. A negative value is indicative of negative emotion, zero is considered neutral, and a positive value indicates positive emotion. All emotional valence values were continuous in nature. Importantly, our aim was not to replicate the results obtained by Yu (2022), but rather to use the VADER package as a first step to test our novel method for classifying sequential emotion within a dream via specific emotion indicators, given that this package has been used successfully in a similar (dream emotion research) context by Yu (2022).

#### 2.7.1 Dream sentiment analysis procedure

We calculated the average word count across dreams, which was 123.289 with a range of 46–202. Regarding the sentiment analysis procedure, firstly, we pre-processed all dream reports to ensure that only *dream descriptions* (reflecting the actual dream experience) as opposed to *dream commentar*y (reflecting thoughts about/interpretations of the dream) were included in each dream report. Following this, we ran each of the 14 pilot dream reports through the VADER package in R. This produced continuous valence ratings for each word in the dream that was either negative, neutral (i.e., 0), or positive in nature. We exported the word-for-word sequential valence ratings for each dream. Next, we applied a “sliding window method” (Martin et al., 2020) as a standardization technique to the dream reports in order to control for two important potential confounds: (1) varying word counts across different dream reports, and (2) difficulties in comparing the degree of emotion across different dreams.

**Figure 1:**
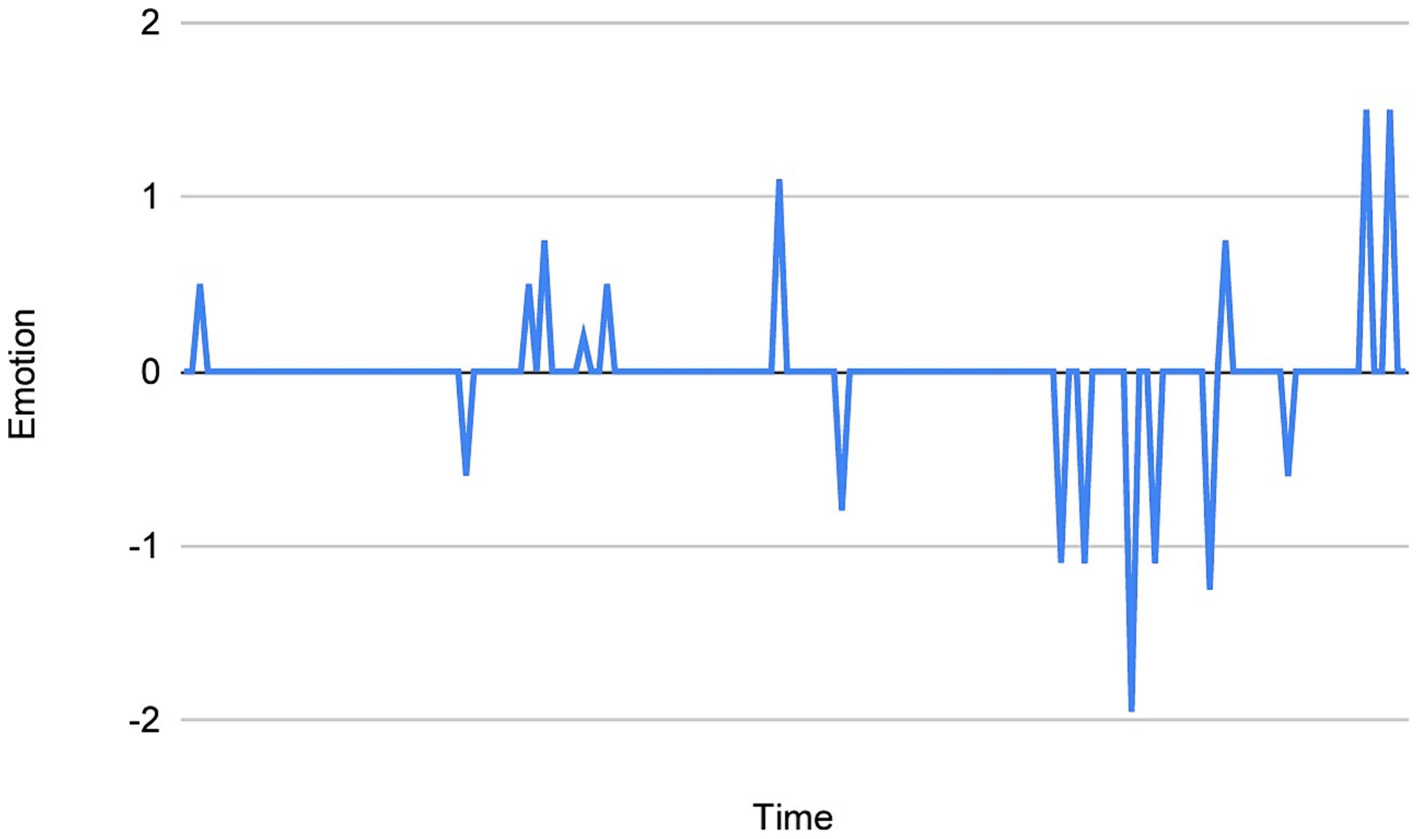
An example of a chart *without* the sliding-window method applied.

With regard to the specifics of this method, it involves dividing each dream report into fixed-length windows, each containing 30 words and overlapping by 29 words. Following this, the emotion/valence ratings of each 30-word window is summed and then divided by the number of windows in the respective dream reports. This method controls for varying word counts across dreams, and importantly, allows us to “extract” the emotion from the dream when plotting the data points. This is because it eliminates the “flattening” effect that many zero values (neutral/absent emotion) in a dream could have on, for example, the trendline of the chart, as well as the “surface area” of the peaks and troughs containing the plotted valence ratings. See Figure 1 (chart without the sliding window method), and 2 (chart with the sliding window method) for comparison.

### 2.8 Generating the emotion indicators

First, we visualized sequential emotion across the dream by plotting the valence ratings in what we call a “dream chart.” Next, we applied statistical and mathematical techniques to the “micro” properties of the dream chart (e.g., the peaks and troughs in the chart), and secondly to the “macro” properties of the dream chart (e.g., trendline gradient) in order to generate the set of emotion indicators that signify the respective micro and macro changes in emotion across a dream.

#### 2.8.1 Peaks and troughs

**Figure 2:**
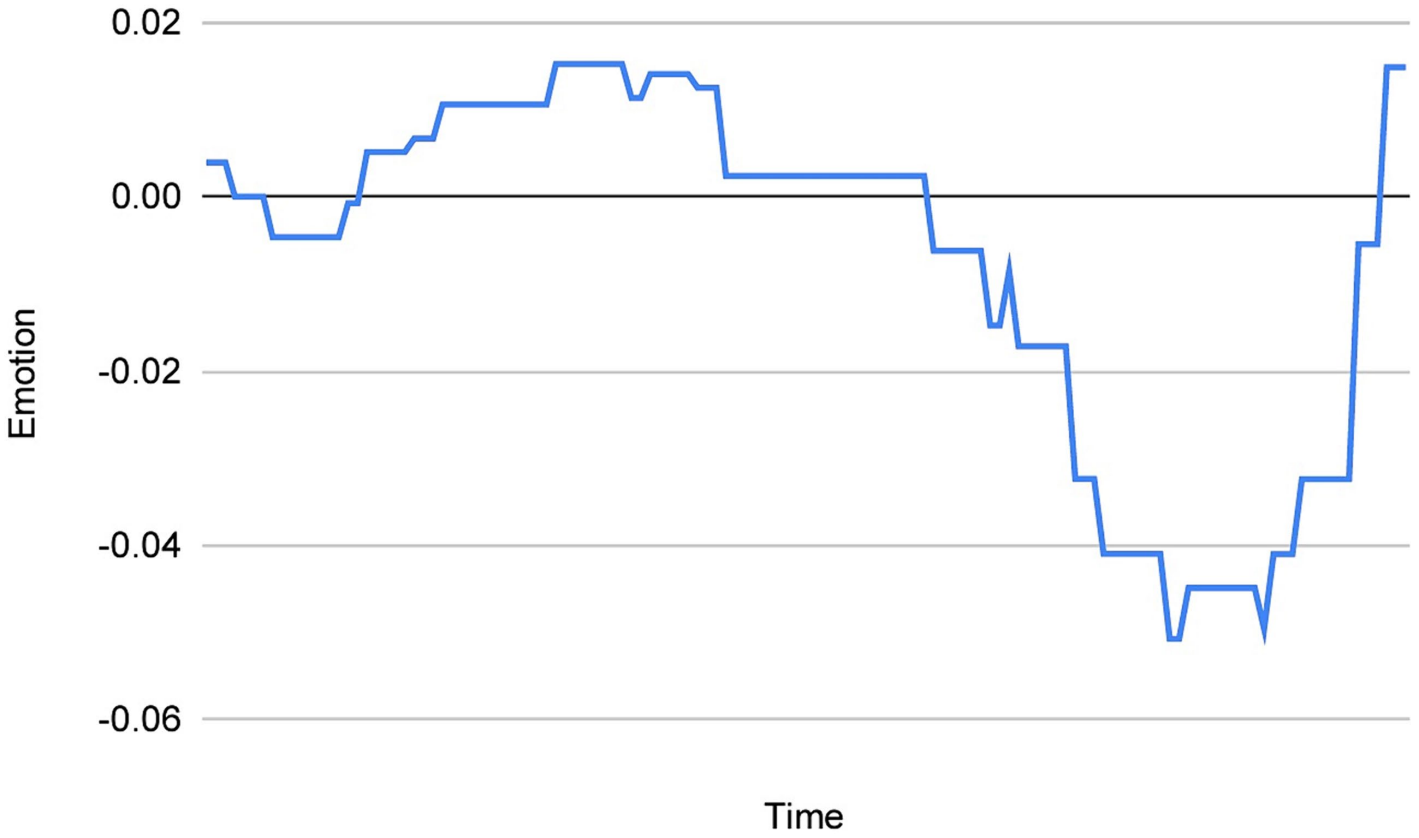
An example of a chart *with* the sliding window method applied.

Positive emotion is represented by peaks in the dream chart and negative emotion is represented by troughs in the dream chart, with the midline being zero (neutral/absent emotion). The first step was to classify/demarcate the peaks and/or troughs contained in each dream chart. Each chart represents the sequential emotion/valence ratings contained in one dream report. The starting point of a peak was defined as the first positive valence value identified above the horizontal line representing 0 (the absence of emotion, see Figure 2), while its end was defined as the first value of 0 following one, or a sequence of positive values. There could be no, a single, or several peaks present in one dream chart. Similarly, a trough was defined as the first negative valence value identified below the horizontal line representing 0 (the absence of emotion), while its end was defined as the first value of 0 following one, or a sequence of negative values. There could be no, a single, or several troughs present in one dream chart. It is theoretically possible for a peak or a trough to consist of a single positive value in the case of the former, and a single negative value in the case of the latter.

##### 2.8.1.1 Emotion indicators based on dream peaks and troughs

We developed a series of primary dream emotion indicators based on the microstructure (peak and trough properties) of the dream charts. Below we discuss each indicator in more detail and provide formulas where applicable.

##### 2.8.1.2 Total peaks and troughs

The first two emotion indicators based on the peak and trough properties in the dream charts include the total number of peaks and troughs present in a single dream chart:


$$\sum_{\mathrm{Peak}}=\mathrm{TotalPeaks}$$

$$\sum_{\mathrm{Trough}}=\mathrm{TotalTroughs}$$

#### 2.8.2 Emotion intensity and area under the curve

We also wanted to determine whether the height of the peaks, or, conversely, the depth of the troughs, are influential parameters when objectively quantifying sequential emotion within a dream. Based on this, we refer to the emotion indicators discussed in this section as the “intensity” of positive and negative emotion within a dream, as well as an “overall emotional intensity” indicator.

Given the small number of dream reports of this pilot study, we have a limited range of chart and curve characteristics to work with when devising and conducting analysis. This coupled with the exploratory, POC nature of this study, as well as the aim of future studies replicating and refining the methods, we decided to implement a simple preliminary method as a starting point to approximate the area under the curve for each peak and trough in the dream charts. Put differently, the aim was to use this method to provide us with an initial approximation of the “emotional real estate” (positive or negative) that each peak and trough occupy on the surface area of the dream chart.

##### 2.8.2.1 The trapezoid rule for approximating peak and trough AUCs

**Figure 3:**
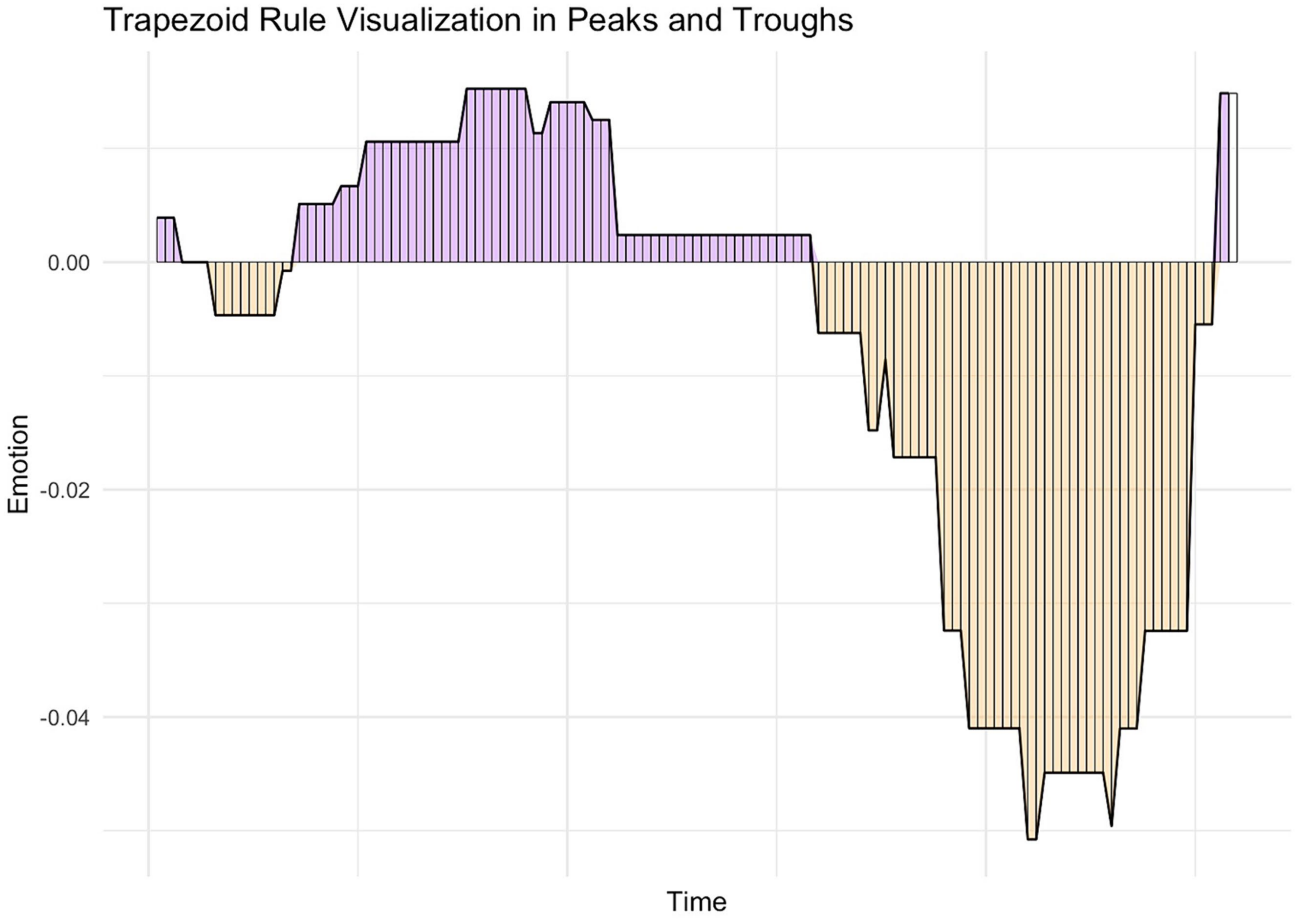
Trapezoid rule visualization in peaks and troughs. Peaks are shaded in purple, and troughs are shaded in orange. Peaks and troughs are segmented into mini trapezoids/rectangles using the Trapezoid Rule visualized by the vertical lines within each peak and trough. The summed segments represent an approximation of the area under the curve for each peak and trough.

The trapezoid rule is an integration rule, in calculus, that evaluates the area under the curve by segmenting the total area into mini trapezoids and summing their areas. We used this method to estimate the area under the curve (AUC) of the respective peaks and troughs contained in our dream charts. In our dream charts, the x-axis represents a “time variable” indicative of the chronological sequence of the dream on a uniformly spaced scale. The y-axis represents the sequential word-for-word emotion ratings derived from sentiment analysis. We used the trapezoidal rule to compute the AUC by approximating each segment between consecutive data points as a trapezoid, and consequently summing their areas (see Figure 3 for a visualization). This computation generates an approximation of the integral of the curve, which represents the cumulative emotionality contained in each peak and trough across the dream chart in a chronological fashion. Put differently, the AUC in this context represents an objective, quantitative indicator of the cumulative positive emotional intensity for each peak, and cumulative negative emotional intensity for each trough. It assists us in uncovering the distribution and intensity of positive and negative emotional intensity within and across a dream.

###### 2.8.2.1.1 Emotion intensity indicators

We derived three composite emotion indicators by applying the trapezoidal rule to calculate the approximated AUC of the peaks and/or troughs contained in our dream charts: (1) positive emotion intensity, (2) negative emotion intensity, and (3) overall emotion intensity.

####### 2.8.2.1.1.1 Positive emotion intensity

This emotion indicator was calculated by summing the approximated AUCs of all the peaks contained in a dream chart (visually represented by all the purple mini trapezoids in Figure 3), which equates to the Positive Emotion Intensity (PEI) indicator. Larger positive values of this indicator denote a higher positive emotion intensity detected in dream charts. In the event where there were no peaks present in a dream chart, the PEI value = 0. In the event where there was only one peak per dream chart, the approximated AUC of the relative peak = PEI. See formula below for calculating the PEI for a dream chart containing >1 peak, where:


PositiveEmotionIntensity=P

Peak = a peak defined by the method outlined in the preceding section

aAUC= approximated area under the curve for each peak per dream chart


$$P=\sum {\mathrm{aAUC}}_{\mathrm{Peak}}$$

####### 2.8.2.1.1.2 Negative emotion intensity

This emotion indicator was calculated by summing the approximated AUCs of all the troughs contained in a dream chart (visually represented by all the orange mini trapezoids in Figure 3), which equates to the Negative Emotion Intensity (NEI) indicator. Larger negative values of this indicator denote a higher negative emotion intensity detected in dream charts. In the event where there were no troughs present in a dream chart, the NEI value = 0. In the event where there was only one trough per dream chart, the approximated AUC of the trough = NEI. See formula below for calculating the NEI for a dream chart containing >1 trough, where:


NegativeEmotionIntensity=N

Trough = a trough defined by the method outlined in the preceding section

aAUC= approximated area under the curve for each trough per dream chart


$$N=\sum {\mathrm{aAUC}}_{\mathrm{Trough}}$$

####### 2.8.2.1.1.3 Overall emotion intensity

This emotion indicator was calculated by summing the following: the aAUCs of all the peaks in a dream chart along with the absolute aAUCs of all the troughs in a dream chart (visually represented by all the orange and purple mini trapezoids in Figure 3). Absolute values were used for troughs in order to capture the full range of emotion intensity of the summed peak and trough aAUCs. Put differently, and conversely, retaining the negative sign of trough aAUC values would have subtracted, as opposed to added, to the range of positive and negative emotional intensity represented by the peaks and troughs in the dream charts. See formula below for calculating the Overall Emotion Intensity, where:


E=OverallEmotionIntensity

p=PositiveEmotionIntensity

n=NegativeEmotionIntensity

|n|=AbsolutevalueofNegativeEmotionIntensity

E=p+|n|

#### 2.8.3 Emotion gradient: dream chart trendlines

In this method, we aimed to use a “macro” emotion indicator, called “emotion gradient,” as a signifier of the overall direction of sequential emotion changes within a dream. In order to achieve this, we used the trendline gradient of the dream charts as the primary macro emotion indicator. This macro indicator stands in contrast to examining the properties of peaks and troughs (and the composites derived from these properties), as micro-level indicators of the direction of emotional changes within a dream. This is consistent with determining, in a preliminary fashion, whether the dream chart trendline gradients could serve as proxies for overall emotion regulation unfolding within the dream.

**Figure 4:**
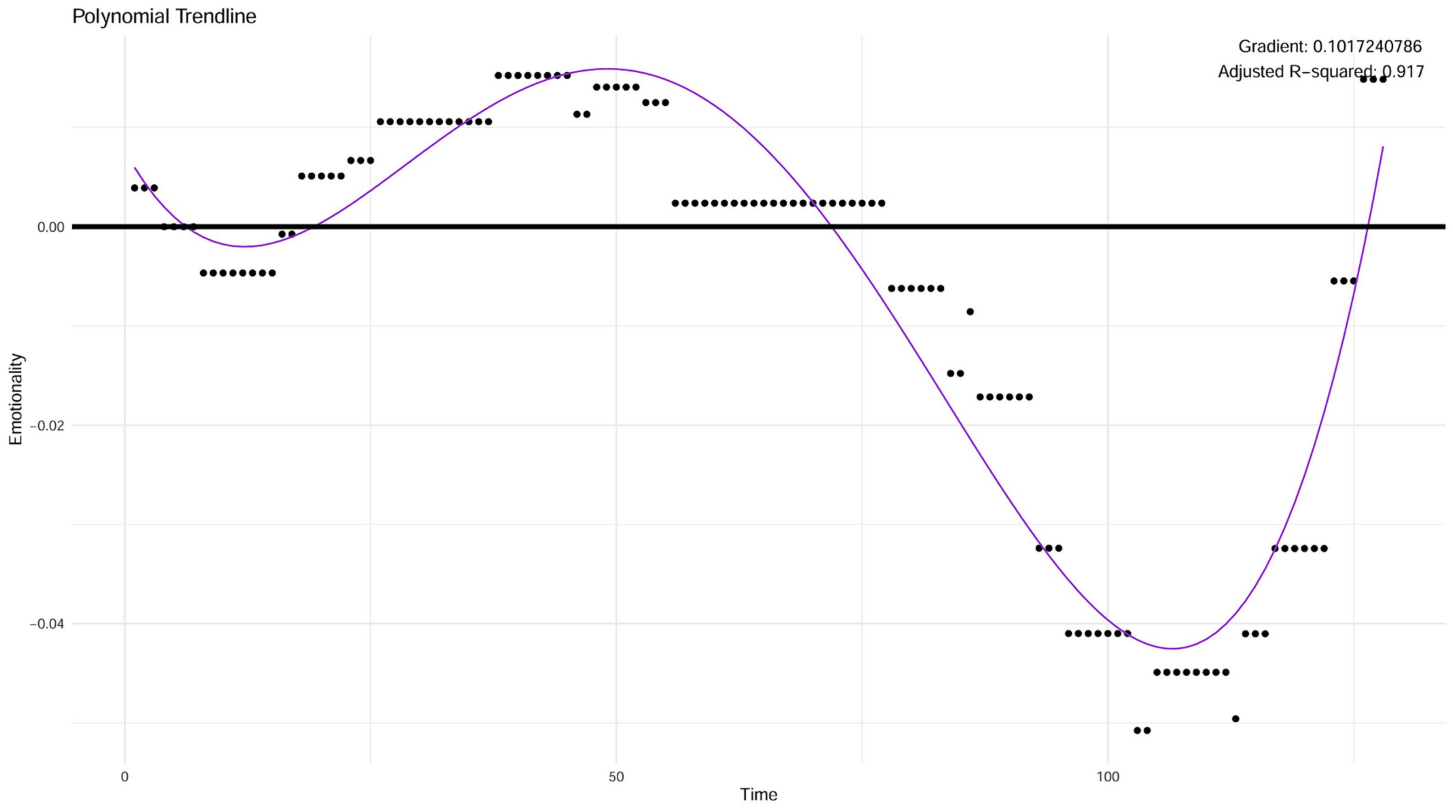
Example of a polynomial trendline fitted to a dream chart.

Given the predominantly fluctuating nature of the data (typically oscillating between peaks and troughs, often with multiple oscillations), preliminary analyses showed that, overall, the most appropriate fit for the data was a polynomial trendline (as opposed to a linear trendline). We decided to use a polynomial trendline as an approximation of the overall/macro direction of emotion changes across a dream. Twelve out of the 14 pilot dream charts exhibited properties where a polynomial trendline would constitute an appropriate fit. Since a polynomial trendline is an appropriate fit for the vast majority of dream charts, we decided to proceed with fitting this type of trendline to the eligible dream charts. Following this, we also examined goodness-of-fit statistics to evaluate the suitability of using a polynomial trendline for all dream charts included in analysis. All analyses were conducted using R statistical software, Version 2023.09.0 + 463. See Figure 4 for a visual example of a polynomial trendline fitted to a dream chart.

The emotion indicator derived from this method is based on the polynomial trendline gradient and is called the “emotion gradient” of dreams. Importantly, again, we propose this method as a theoretical starting point for future studies to replicate and refine.

### 2.9 Statistical analysis

All data was analyzed using R statistical software, Version 2023.09.0 + 463. We included two sets of variables in the correlational analyses: “emotion indicators” (the novel variables described in the preceding sections), and “external variables” (existing sleep, state, and trait variables recognized to have a relationship with dreaming and/or emotion regulation). Descriptive statistics indicated that the variables typically exhibited non-linear properties. This is likely, at least partially, due to the small sample size included in this pilot study. Due to the distribution properties of the data, we decide to run Spearman’s Rho, as a non-parametric alternative, provided there were no ties present in the data (Spearman’s rho ranks the data, and ties can distort true results). In the event where there were ties present in the data for a particular variable, Pearson’s R was used as an alternative. We readily acknowledge that this is not an ideal solution; however, due to the exploratory and POC nature of this pilot study, we decided to proceed and report any significant correlations found, especially if there is existing empirical evidence to support the relationship between the emotion indicators and the external variables. Furthermore, because of the small sample and our intention to provide pilot data, we did not correct for multiple correlations.

## 3 Results

### 3.1 Emotion indicators and external variables

The final emotion indicators included the following: Total Peaks (Peaks), Total Troughs (Troughs), Positive Emotion Intensity (PEI), Negative Emotion Intensity (NEI), Overall Emotion Intensity (OEI), and Emotion Gradient (Emo_Grad). The external variables included in analyses can be divided into three broad classes: (1) PSG-derived sleep variables (classified as “sleep” variables), which include variables like sleep stage distribution and number of awakenings, (2) affective variables (classified as “state” variables), which are variables derived from the Positive and Negative Affect Scale—Extended Version (PANAS), and (3) personality variables (classified as “trait” variables) derived from the Ten-Item Personality Inventory, and the Boundary Questionnaire.

In relation to our first and second objectives, generating emotion indicators from quantifying and visualizing emotion fluctuations using sentiment analysis, we ran descriptive statistics on all the micro- and macro-level emotion indicators.

These results show that we were able to successfully classify and quantify the fluctuations between positive and negative emotions within a dream using the sentiment analysis package, VADER, in R (see Table 1). The directions of these fluctuations were largely consistent with the emotional tone of the dream as subjectively evaluated by the researchers.

**Table 1:** Descriptive statistics for emotion indicators derived from sentiment analysis.

Emotion indicator	Mean (SD)	Range
Peaks	1.57 (1.16)	4.00
Troughs	1.14 (1.10)	3.00
Positive emotion intensity	1.04 (1.12)	3.76
Negative emotion intensity	−0.60 (0.85)	2.16
Overall emotion intensity	1.59 (1.14)	4.20
Emotion gradient	0.012 (0.037)	0.14
Word count*	123.289 (59.689)	46–202

Peaks = average number of Peaks present per dream report across all dreams (*N* = 14). Troughs = average number of Troughs present per dream report across all dreams (*N* = 14). Positive Emotion Intensity = the average approximated area under the curve for all Peaks in a dream across all dreams (*N* = 14). Negative Emotion Intensity = the average approximated area under the curve for all Troughs in a dream across all dreams (*N* = 14). Overall Emotion Intensity = the summed Positive Emotion Intensity and the absolute Negative Emotion Intensity for each dream across all dreams (*N* = 14). Emotional Gradient = the average of the overall direction of sequential emotion changes within a dream across all eligible dreams (*N* = 11). *We have used the sliding window method to control for discrepancies in word counts across dreams.

**Figure 5:**
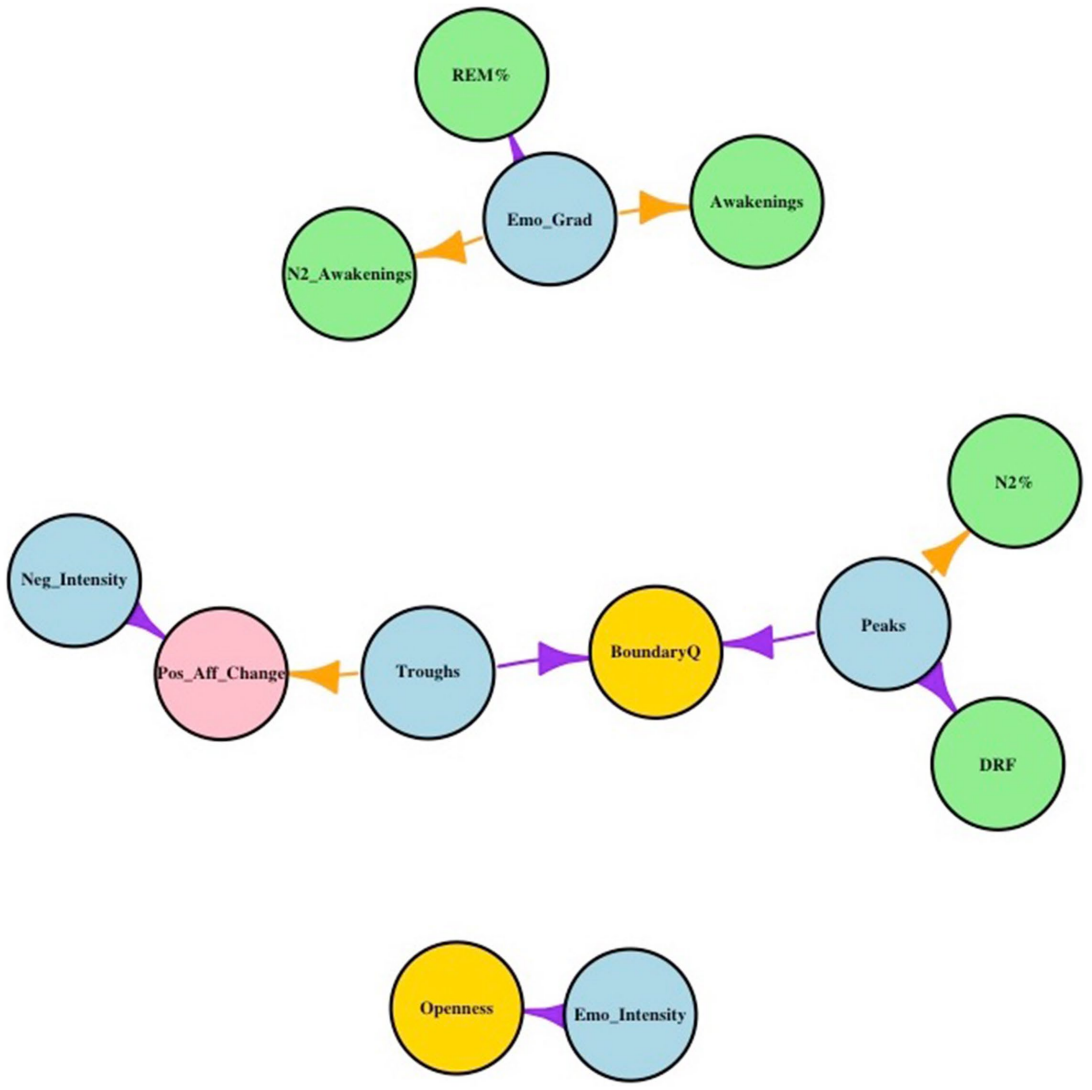
All emotion indicators are presented in light blue: Peaks = Total Peaks; Troughs = Total Troughs; Emo_Fluct = Emotion Fluctuations; Neg_Intensity = Negative Emotion Intensity; Emo_Intensity = Overall Emotion Intensity (OEI); Emo_Grad = Emotion Gradient. The state variable is presented in pink: Pos_Aff_Change = percentage overnight change in positive affect. Trait variables are presented in yellow: Openness = TIPI-Openness Big 5 Personality dimensions; BoundaryQ = Boundary Questionnaire—Shortform (higher score = “thinner” boundaries). Sleep variables are presented in light green: Awakenings = The total number of awakenings across the night; N2_Awakenings = Awakenings from stage 2 non-REM sleep across the night; REM% = percentage of time spent in REM sleep across the night; N2% = percentage of time spent in non-REM stage 2 sleep across the night; DRF = dream recall frequency. Purple arrows denote a statistically significant *positive* relationship between an emotion indicator and external variable(s), while orange arrows denote a statistically significant *negative* relationship between an emotion indicator and external variable(s).

With regard to our second objective, correlating the above-mentioned emotion indicators with sleep, state, and trait variables, results showed the following: Statistically significant correlations between 5/6 of the novel objective emotion indicators and at least one external variable (sleep, state, or trait variables). The network diagram depicted in Figure 5 visualizes the statistically significant relationships between two sets of nodes: emotion indicators and the different classes of external variables. The direction of the relationships between the two sets of nodes (significant positive or significant negative correlation) are presented as edges in the diagram. The statistical parameters of the results can be found in Table 2.

**Table 2:** Statistical parameters of significant correlations depicted in the network diagram.

	REM%	N2%	Awakenings	N2 awakenings	DRF	Positive affective change	Boundary Q	Openness
Peaks	0.238	−0.548*	−0.269	−0.320	0.608*	−0.307	0.642*	−0.118
Troughs	0.205	−0.529	−0.076	−0.138	0.358	−0.590*	0.702**	−0.097
Negative emotion intensity^ρ^	0.043	0.074	0.052	−0.043	0.103	−0.666**	0.482	−0.047
Overall emotion intensity	0.088	−0.042	0.227	0.141	0.336	−0.179	0.377	0.548*
Emotion gradient^ρ^	0.627*	−0.555	−0.743**	−0.749**	−0.176	−0.536	−0.141	−0.397

Peaks = Total Peaks; Troughs = Total Troughs; REM% = percentage of time spent in REM sleep across the night; Awakenings = The total number of awakenings across the night; N2_Awakenings = Awakenings from stage 2 non-REM sleep across the night; N2% = percentage of time spent in non-REM stage 2 sleep across the night; DRF = dream recall frequency; Positive Affective Change = percentage overnight change in positive affect; Openness = TIPI-Openness Big 5 Personality dimensions; Boundary Q = Boundary Questionnaire—Shortform (higher score = “thinner” boundaries). *p = <0.05; **p = <0.01. ^ρ^ = Spearman’s rho is reported where there were no ties present in the dataset, else Pearson’s r is reported.

With regard to the first emotion indicator, the emotion gradient, the results show, firstly, that a *steeper, positive* emotion gradient in a dream chart has a significant *positive* relationship with the proportion of REM sleep a person had across the night. Put differently, a *steeper upward* trend of positive emotion across a dream is associated with an *increased* amount of REM sleep across the night. Secondly, results show that a *steeper, upward* trend in positive emotion across a dream is significantly associated with *fewer* overall awakenings, and awakenings from N2, across the night.

The next observed trend involves two emotion indicators based on the microstructure of the dream chart—trough characteristics: the results show that (a) fewer troughs across the dream chart, and (b) troughs with a larger surface area (AUC), are significantly associated with a *higher percentage increase* in overnight positive affect. Therefore, the properties (number and size) of dream troughs appear to be important in affective processes (state variable) in this sample. In addition, the number of troughs in a dream chart is also significantly associated with having “thinner” boundaries (“trait” variable) as measured by the Boundary Questionnaire. Interestingly, the number of peaks, another indicator of the microstructure of the dream chart, is also significantly associated with having “thinner” boundaries. However, where the number of throughs showed a significant association with affective processes (state variable), the number of peaks shows an additional significant association with two sleep variables: N2% and DRF. More specifically, a *higher* number of peaks present in a dream chart is significantly associated with (a) having *less* overall N2 sleep across the night, and (b), having *higher* dream recall rates.

The third and final trend derived from the network diagram relates to another indicator of the microstructure of the dream charts—the overall emotion intensity (OEI) indicator. The OEI is a composite measure that is based on the summed approximated AUCs of all the peaks and troughs present in the dream chart. Therefore, the overall emotion intensity of a dream is influenced by both the number of peaks and troughs, as well as the height and depth (intensity) of the respective peaks and troughs. In summary, it is a composite of the approximated positive and negative intensity of emotion contained in each dream chart. The results show that there is a significant association between higher emotion intensity and scoring high on the “Openness” dimension of the Big Five personality dimensions as measured by the TIPI. Put differently, participants with a higher receptivity to new ideas and experiences tended to have higher overall emotion intensity in their dreams in this sample.

## 4 Discussion

In this study, we aimed to develop a novel method for objectively classifying and quantifying sequential emotion fluctuations within a dream. We propose that these fluctuations could serve as a proxy for emotion regulation processes unfolding within a dream. We used sentiment analysis, a branch of Natural Language Processing (NLP), as the objective measure for assigning emotion valence ratings to each word in a dream report. Next, we implemented a sliding window method (Martin et al., 2020) in order to (a) control for varying word counts across dream reports, and (b) for “extracting” emotion from the dream reports by eliminating the flattening effect that many 0 s (neutral emotion) could have on the peak and trough properties, as well as the trendline gradient of the dream charts. Based on these results we generated 6 “emotion indicators” derived from both the micro- and macro-level properties of the dream charts. Finally, we correlated the emotion indicators with three classes of external variables: sleep, state, and trait variables.

We were able to successfully complete all of the steps summarized above, while results also confirm that we have met our three operationalized objectives: (1) using sentiment analysis to objectively classify, quantify, and visualize sequential fluctuations between positive and negative emotions across a dream, (2) using these visual properties along with the valence ratings derived from sentiment analysis to generate “emotion indicators,” and (3) preliminarily validating the novel emotion indicators by showing a statistically significant correlation with external variables (sleep, state, and trait) known to be associated with dreaming and/or emotion regulation.

### 4.1 Limitations and directions for future research

The first limitation of this pilot study relates to not including the ratings of several independent, expert (human) raters of dream reports. We suggest that future studies incorporate this into their methodology as an external form of validation with regard to the valence ratings generated by sentiment analysis. The second limitation of this pilot study relates to the small sample size and the limitations this imposes on the results. Therefore, we present our findings in this methodological paper as preliminary and as a proof-of-concept for future studies to replicate and refine. More specifically, we propose that future studies recruit (a) larger, and (b) more diverse (e.g., clinical and-nonclinical) samples. Next, we propose that future studies test the trapezoid rule as a method for approximating the area under the curve against other, potentially more precise and refined methods. In addition, we suggest future studies explore additional types of trendlines to be fitted based on the specific properties of their dream charts so that different types of dream charts can be accommodated in the sample in its entirety. Generating additional types of emotion indicators is another avenue for future studies to pursue. Finally, although beyond the scope of this methodological article, future studies should aim to build on our results from correlational analyses in order to elucidate the nature of the relationship between the emotion indicators and the sleep (e.g., REM%), state (e.g., overnight affective change), and trait (personality) variables. This could be done, for example, by experimentally testing the proposition that these (or other) emotion indicators serve as proxies for emotion regulation processes unfolding within a dream. Investigating dream-related emotion regulation processes has important implications, not only for garnering insights into the course of healthy dreaming, but for understanding psychiatric disorders, where emotion dysregulation and alterations in dream activity coincide.

## 5 Conclusion

In this proof-of-concept paper we have described a novel method for objectively quantifying sequential emotion within a dream using sentiment analysis. We also present preliminary evidence from descriptive statistics and correlational analyses that support the rationale for using sentiment analysis in this manner, as well as in support of the (preliminary) validity of the emotion indicators derived from our method. We see this method along with the accompanying results as a first step in developing a new, objective method for rating and visualizing emotion in dreams. We also propose that the emotion indicators we have developed could serve as potential proxies for emotion regulation processes unfolding during a dream. With these preliminary findings we aim to provide a methodological foundation for future studies to test and refine the method employed in the current study in larger and more diverse samples.

## Figures and Tables

**Figure A1:**
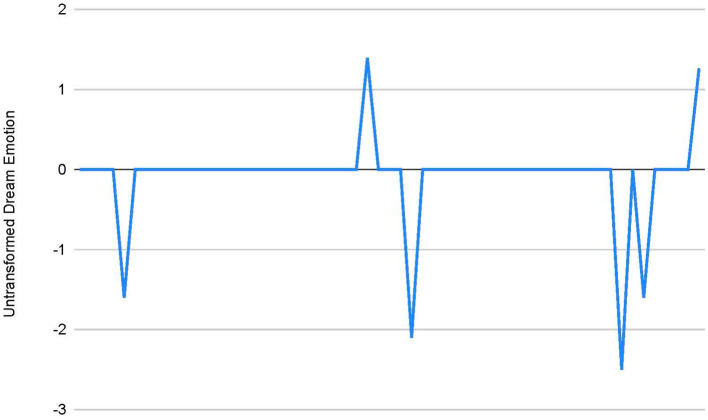
Visualization of untransformed dream emotion prior to applying the sliding window method in Dream 1.

**Figure A2:**
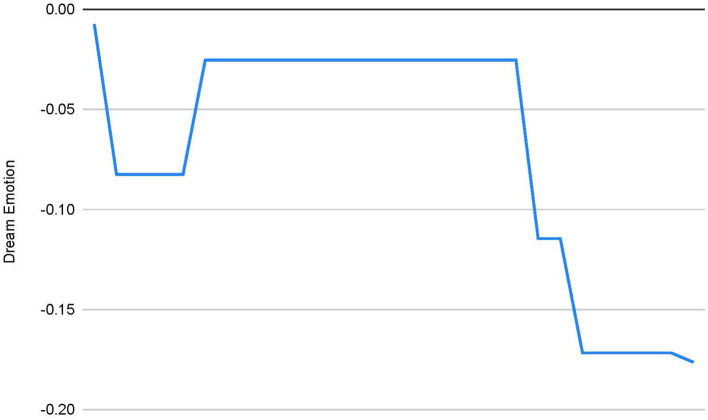
Visualization of dream emotion after applying the sliding window method in Dream 1.

**Figure A3:**
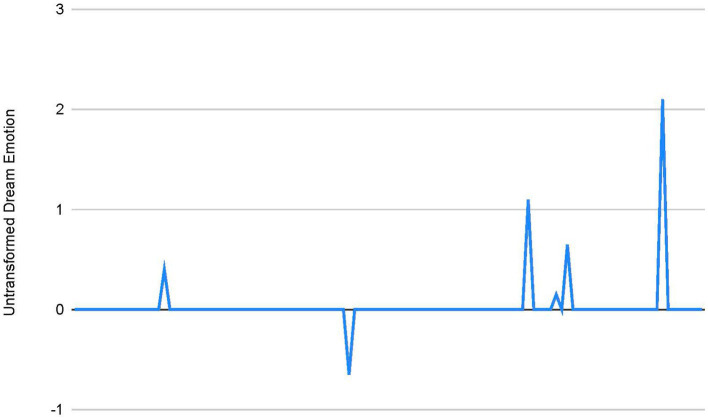
Visualization of untransformed dream emotion prior to applying the sliding window method in Dream 2.

**Figure A4:**
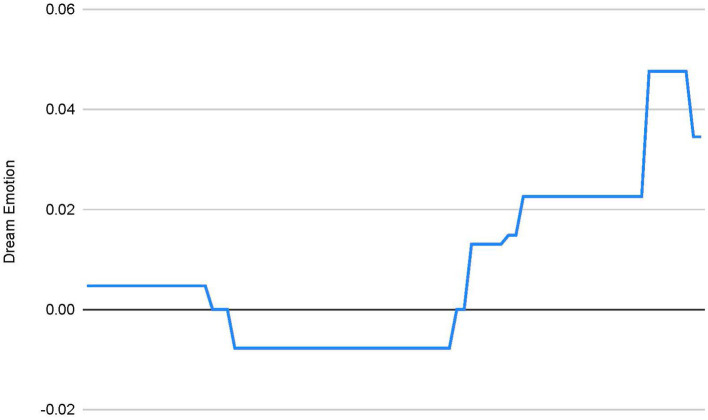
Visualization of dream emotion after applying the sliding window method in Dream 2.

## Data Availability

The raw data supporting the conclusions of this article will be made available by the authors, without undue reservation.

## Appendix

Dream report example 1:

*My boyfriend’s mother was upset with me. We were at his house, in his kitchen. We were making samoosas and rotis and I wasn’t doing very well. I ended up crying because she said that we could not get married because my rotis were not round enough and my samoosas are bad. I cried for most of my dream.*

See Figure A1 for visualized valence ratings from sentiment analysis using the VADER package *prior to* applying the sliding window method.

See Figure A2 for visualized valence ratings from sentiment analysis using the VADER package *after* applying the sliding window method.

Dream report example 2:

*I was at Comic Con and I was leaving at one of the gates and Taylor Swift, Selena Gomez and Taylor’s girlfriend were standing outside it. I was waiting for someone to come fetch me so I was on my phone. Taylor leaned forward and said “hello.” I was shocked and said “hi” and introduced myself and we spoke about where I came from* etc. *and then Taylor invited me on her next tour with her. When I went home, my friend Matthew (who is Taylor’s number 1 fan) was there. I started making pasta while telling him about my experience but I was too excited, so he made my pasta for me.*

See Figure A3 for visualized valence ratings from sentiment analysis using the VADER package *prior to* applying the sliding window method.

See Figure A4 for visualized valence ratings from sentiment analysis using the VADER package *after* applying the sliding window method.
